# Albany County Medical Society, Semi-Monthly Meeting

**Published:** 1873-03

**Authors:** F. C. Curtis

**Affiliations:** Secretary


ART. III.—*Medical Society of the County of Albany, Semi-Monthly Meeting*, Feb. 25th, 1873. Reported by F. C. CURTIS, M. D. Secretary.

According to a vote of the Society, a discussion of the subject of Paracentesis of the Thorax, on which Dr. E. H. Davis read a paper at the last meeting, was the first in order.

Dr. HENRY MARCH reported eleven cases in which his father, the late Dr. Alden March, had performed the operation. In five of them he operated for the purpose of affording temporary relief only, the patients being affected with tuberculosis and cancer, of which they subsequently died. We may infer that in his opinion we are justified in operating under these circumstances. Two of the subjects were children of seven and nine years. In all the cases he used the trocar.

Dr. M. M. LAMB said that six months ago he undertook the treatment of a child who a year before had passed through an attack of Scarlatina, since which it had never been well. There had been a chronic diarrhœa and cough and emaciation. He found it wasted away, having dyspnoea and with cavities in the right lung. Some time after a tumor appeared at the fourth interspace on the right side, which proved to be a pointing abscess. He opened it with a scalpel and discharged a quantity of pus. The child now began to improve rapidly, gaining in flesh and strength. The discharge continued but grew less in amount, until it has now ceased almost entirely, and the child at the present time, three months after the opening of the abscess, is very robust although the lung is so seriously diseased.

Dr. JOSEPH BLATNER read a translation on the subject under consideration from a work of the late Professor Oppolzer of Vienna. After detailing the principles which should govern the medical treatment, he next spoke of the indications for paracentesis. It should be undertaken, he says, when the amount of the effusion, or the resulting oedema of the lungs threatens suffocation, other means, especially venesection, having been previously tried in vain. Such a condition existing, it may be performed both in chronic and acute pleurisy and also in phthisis. It is indicated when the effusion has stood for several weeks without decrease, being so large as to obstruct absorption on account of the pressure on the blood and lymph vessels of the pleura. The dangers of allowing this condition to continue are the occurrence of a deadly swoon, wasting of the lung so that it cannot again develop itself, emphysema, phthisis, rigidity of the chest walls, etc.

When the effusion is purulent, operation is particularly indicated. On this all authorities agree, even when the effusion is a consequence of pyæmia. More especially are we called upon to open the pleural sac when the lung is perforated and we have pyopneumothorax, for here there is great danger of septic poisoning. The same is true of a confined purulent effusion causing a spontaneous generation of air.

For the operation the trocar is preferred for simple serous effusion, the canula having an arrangement to hinder the ingress of air. But if it is purulent the knife should be used and a fistula established, closure being prevented by tents or an elastic catheter. Experience teaches, in regard to the fistula, that a single puncture seldom effects a cure in empyæma, and if allowed to close a spontaneous fistula sooner or later is established. The entrance of air must necessarily ensue, but this in a purulent effusion can cause no harm if the fluid is carefully withdrawn. It should be syringed out twice daily with luke warm water to which a solution of permanganate of potash or common salt has been added. By paracentesis alone it is not possible to withdraw all the fluid, and while if this is simple serum it will generally be absorbed, if it is pus it will, even if air is excluded, give rise to fresh inflamation and effusion. In all these cases the patient must be supported with quinine and a proper diet. If paracentesis is performed for a non-purulent effusion it is recommended to take away a portion only, for these reasons: if the pressure is taken off the vessels too suddenly fresh exudation is likely to follow; experience teaches that the part that is left will be absorbed.

As to the point of operating, the walls should be pierced as a rule in the 4th or 5th interspace, in a direction forward of the axilla, unless abnormal attachment of the heart or lungs forbid. A horizontal position is preferred during the operation on account of the tendency to faint.

Dr. Van Derveer spoke of the method of treating empyæma by means of establishing a fistula. This is the way in which nature deals with it when it effects a cure. He spoke of eleven cases of gun shot wounds of the chest which he had seen in the army. In nine of these empyæma occurred. The pus discharged freely from one or both openings made by the ball. All but two made good recoveries. He believed that the danger, ordinarily taught and believed, of entering the cavity of the pleura or allowing air to enter it is much exaggerated.

Dr. Hailes reported a case occurring in the Hospital of empyæma following pleuro-pneumonia, for which the chest was tapped. A fistula followed which lasted for some time and finally closed, but a repetition of the symptoms called for a second tapping. The pleural cavity was then washed out with water to which a little salt and carbolic acid were added by means of a gum catheter and Davidson's syringe. He subsequently died of tuberculosis. He spoke of Dieulafoy's Aspirator which he believed would prove a valuable means in certain cases for removing these accumulations.

Dr. James S. Bailey presented a case of Lipomata or Fatty Tumors of the Abdomen with the pathological specimens. After giving a sketch of the literature of the subject, he gave a history of the case which was meagre as he never saw the patient during life. The subject, a woman aged 69 at the time of her death, began 12 years ago to experience a heavy feeling in the abdomen, as if a menstrual period was about to begin. A tumor appeared low down on the left side and grew in size slowly until it included the whole area of the abdomen; no pain attended except what might be attributed to pressure on surrounding viscera. Probably from the same cause she had irregularity in the action of the bowels, a period of constipation being followed by a watery diarrhœa. She was confined to bed for six months prior to death, failing slowly. At the autopsy the abdomen was found uniformly enlarged, projecting abruptly over the brim of the pelvis and reaching up so as to bulge out the four lower ribs. This was found on section to be due to two lipomata lying directly under the walls of the abdomen and occupying most of the cavity, having broad and slightly defined attachments at the posterior ligaments of the uterus. They weighed respectively 15 and 22 pounds. They were covered with peritoneum by which they must have been nourished in good part as they were but slightly vascular. A sister and daughter of the subject are affected in the same way.

Dr. Bailey also reported a case of *Meningocele* which occurred in the practice of Dr. M. M. Lamb. The subject of the malformation was a male child, one of twins, and weighed eight pounds at birth. Labor was tedious but not difficult. There was a tumor one third larger than the head, emerging from the posterior fontanelle, and having a covering similar to the scalp. It was soft to the touch. Internally it was filled with sub-arachnoid fluid enclosed in the membranes of the brain. A similar case is reported and shown in the Photographic Review of Philadelphia for 1870—71. The child nursed well but died on the 14th day.

Dr. Van Derveer presented a case of Cerebral Apoplexy, remarkable for the extent of the injury which it did to the brain. The subject was a man aged about 60. He had been a fast liver and a free drinker. For a year back his life had been very sedentary.

Four days before his death, his health being up to the time as good as usual, he was taken in the night with marked symptoms of apoplexy, and never returned to consciousness.

The following is the report of the autopsy made 24 hours after death.

*External appearances:* Rigor mortis well marked; Body well developed; Skin tinged slightly yellow.

*Abdomen:* Liver somewhat enlarged, edges rounded and it was fatty. The under surface was firmly adherent to a portion of the omentum and small intestine, the gall bladder being included in the mass and entirely collapsed. Kidneys—both large, fatty and granular. The left was especially affected, the pyramids being obliterated. The bladder was empty of urine.

*Thorax:* The Lungs were healthy. The heart contained a small fibrinous clot in the left ventricle. Aortic and mitral valves ossified.

*Encephalon*: Clear serous effusion over the surface of the brain under the arachnoid. Spots of gummatous deposit were on the membrane. The vessels were congested. Brain on section firm. On the right side a large clot filled a space of a size large enough to admit a medium sized apple. This was in the central portion of the middle lobe of the right hemisphere, extending from the lateral ventricle, which was torn into, to the surface. The left lateral ventricle also contained bloody serum but no clot. The vessels of the brain were atheromatous, the basilar artery being completely calcified. The hemmorrhage was probably due to rupture of a branch of the middle cerebral artery.

